# Potential role of immunotherapy and targeted therapy in the treatment of cancer: A contemporary nursing practice

**DOI:** 10.1016/j.heliyon.2024.e24559

**Published:** 2024-01-17

**Authors:** Hamad Ghaleb Dailah, Abdullah Abdu Hommdi, Mahdi Dafer Koriri, Essa Mohammed Algathlan, Syam Mohan

**Affiliations:** a Research and Scientific Studies Unit, College of Nursing, Jazan University, Jazan, 45142, Saudi Arabia; b Substance Abuse and Toxicology Research Centre, Jazan University, Jazan, Saudi Arabia; c Center for Global Health Research, Saveetha Medical College and Hospitals, Saveetha Institute of Medical and Technical Sciences, Saveetha University, India; d School of Health Sciences, University of Petroleum and Energy Studies, Dehradun, Uttarakhand, India

**Keywords:** Cancer, Immunotherapy, Oncology, Targeted therapy, Nursing practice, Adverse events

## Abstract

Immunotherapy and targeted therapy have emerged as promising therapeutic options for cancer patients. Immunotherapies induce a host immune response that mediates long-lived tumor destruction, while targeted therapies suppress molecular mechanisms that are important for tumor maintenance and growth. In addition, cytotoxic agents and targeted therapies regulate immune responses, which increases the chances that these therapeutic approaches may be efficiently combined with immunotherapy to ameliorate clinical outcomes. Various studies have suggested that combinations of therapies that target different stages of anti-tumor immunity may be synergistic, which can lead to potent and more prolonged responses that can achieve long-lasting tumor destruction. Nurses associated with cancer patients should have a better understanding of the immunotherapies and targeted therapies, such as their efficacy profiles, mechanisms of action, as well as management and prophylaxis of adverse events. Indeed, this knowledge will be important in establishing care for cancer patients receiving immunotherapies and targeted therapies for cancer treatment. Moreover, nurses need a better understanding regarding targeted therapies and immunotherapies to ameliorate outcomes in patients receiving these therapies, as well as management and early detection of possible adverse effects, especially adverse events associated with checkpoint inhibitors and various other therapies that control T-cell activation causing autoimmune toxicity. Nurses practice in numerous settings, such as hospitals, home healthcare agencies, radiation therapy facilities, ambulatory care clinics, and community agencies. Therefore, as compared to other members of the healthcare team, nurses often have better opportunities to develop the essential rapport in providing effective nurse-led patient education, which is important for effective therapeutic outcomes and continuance of therapy. In this article, we have particularly focused on providing a detailed overview on targeted therapies and immunotherapies used in cancer treatment, management of their associated adverse events, and the impact as well as strategies of nurse-led patient education.

## Abbreviations

AEs: Adverse events
ALL: Acute lymphoblastic leukemia
AML: Acute myeloid leukemia
aRCC: Advanced renal cell carcinoma
ASM: Aggressive systemic mastocytosis
BCC: Basal cell carcinoma
CAR: Chimeric antigen receptor
cHL: Classical hodgkin lymphoma
CLL: Chronic lymphocytic leukemia
CML: Chronic myelogenous leukemia
CRC: Colorectal cancer
CTCL: Cutaneous T-cell lymphoma
DCs: Dendritic cells
DTC: Differentiated thyroid cancer
ESCC: Esophageal squamous cell carcinoma
FL: Follicular lymphoma
gBRCAm: Germline BRCA-mutated
HCC: Hepatocellular carcinoma
HER2: Human epidermal growth factor receptor 2
HFSR: Hand foot skin reaction
HNSCC: Head and neck squamous cell cancer
HR: Hormone receptor
HTN: Hypertension
ICIs: Immune checkpoint inhibitors
IDO: Indoleamine 2,3-dioxygenase
IL-2: Interleukin-2
LBCL: Large B-cell lymphoma
MAPK: Mitogen-activated protein kinase
MASCC: Multinational association of supportive care in cancer
MCL: Mantle cell lymphoma
mCRC: Metastatic colorectal cancer
mNSCLC: Metastatic non-small cell lung cancer
MoAbs: Monoclonal antibodies
MOATT: MASCC Oral Agent Teaching Tool
MSI-H: Microsatellite instability-high
mTOR: Mammalian target of rapamycin
NHL: Non-Hodgkin lymphoma
NSCLC: Non-small cell lung cancer
PMBCL: Primary mediastinal B-cell lymphoma
PNET: Pancreatic neuroendocrine tumors
PPES: Palmar-plantar erythrodysesthesia syndrome
PTCL: Peripheral T-cell lymphoma
PTEN: Phosphatase and tensin homolog
RCC: Renal cell carcinoma
SCLC: Small cell lung cancer
SLL: Small lymphocytic lymphoma
SM-AHN: Systemic mastocytosis with associated hematological neoplasm
STS: Soft tissue sarcoma
TKIs: Tyrosine kinase inhibitors
URI: Upper respiratory infection
UTI: Urinary tract infection

## 1 Introduction

The term cancer involves numerous different types of malignancy that share some common features including abnormal cells that exhibit uncontrolled cell division and can evade apoptosis or programmed cell death [1]. Cancer is a leading public health problem around the world and tens of millions of people are diagnosed with cancer each year worldwide [2]. After cardiovascular diseases, cancer is the second leading cause of death in many countries. Furthermore, it has been reported that the number of deaths and new cases of cancer will continue to increase because of the increasing aging population and the adoption of lifestyle behaviors that elevate cancer risk [3]. Over the past half-century, treatment options for cancer have advanced from a highly toxic, single chemotherapeutic drug (for example-nitrogen mustard) to targeted therapies that interrupt specific signaling mechanisms and are more tolerable as compared to conventional anticancer drugs. Moreover, current cancer therapies include immunotherapy, biotherapy, adjuvant chemotherapy, surgery, combined modalities including concurrent chemotherapy and radiation or sequential neoadjuvant chemotherapy, and combinations of drugs [4]. Unfortunately, still chemotherapy resistance and recrudescence remain a great challenge [5]. Therefore, novel therapies are urgently needed to treat cancer [6].

The immune system is capable of detecting non-self versus self, thus providing protection to the human body from diseases of endogenous and exogenous origins. The immune system involves white blood cells and lymphoid tissues and organs, such as bone marrow, lymph vessels, lymph nodes, tonsils, spleen, and thymus, which eventually detects many threats and eradicates them to maintain homeostasis. A comprehensive knowledge of the interaction between the immune system and cancer cells is important to appreciate the important role of immunotherapy in cancer therapy. Chemotherapy kills cancer cells through their cytotoxic activities, whereas immunotherapies utilize the host immune system to destroy tumor cells [7]. On the other hand, targeted therapies specifically attack various targets in the cell that consequently interfere with a growth pathway. Improvements in genomic sequencing technology and molecular biology have resulted in the detection of numerous therapeutic targets within cancer cells and the development of therapies to interfere with them. It has been reported that these novel therapies possess fewer toxic side effects owing to their capacity to act precisely on precise targets in the growth pathways with minimum collateral injury to healthy tissues and cells [4].

Chemotherapy or cytotoxic therapy is a traditional or conventional mode of cancer treatment, however chemotherapy is now not regarded as a mandatory therapeutic option for several malignancies. Chemotherapy also involves collateral damage to healthy cells. As compared to chemotherapy, very little cytotoxic side-effects are observed with immunotherapy and targeted therapy [8]. Nurses are the interface between physicians and patients, therefore it is crucial that they have a comprehensive understanding of how newer anticancer drugs affect patients and patient care. There are two major factors that need to be carefully considered in the case of any anticancer drug including adherence to treatment and proper understanding of the adverse events (AEs) of a therapy. The knowledge regarding the AEs of therapy includes an understanding of the management of each AE and assisting effective care coordination. Various other factors that need to be considered involve current medications, co-morbid factors, the patient's performance status, patient suitability for the anti-cancer agent, and the timing of drug administration with respect to consumption of/interaction with food. All these aforesaid factors can affect the efficacy of the anticancer drugs or the capacity of the patients to deal with AEs. In addition, effective management of AEs might ameliorate the health-associated quality of life of patients and maximize their responsiveness to therapies. Patient education is another crucial competency offered by nurses to patients. The definition of patient education includes counseling and training patients [9]. Indeed, patient education is important for the exchange of knowledge and for the active participation of patients in the health care system. Oncology nurses are responsible for providing treatment to a wide range of patients with varying levels of health literacy. Thus, nurses need to utilize specific approaches to confirm useful patient education and techniques that are customized to the patient's preferred method of teaching and level of health literacy. This article provides an overview of the potential role of immunotherapy and targeted therapy as promising therapeutic options for cancer patients. Moreover, this article has broadly covered the role of nursing assessment and management of adverse events in cancer treatment with immunotherapies and targeted therapies, strategies of nurse-led patient education in immunotherapies and targeted therapies, and challenges of combining immunotherapy and targeted therapy.

## 2 Search strategies

A search was performed in Embase Cochrane Central, Google Scholar, Web of Science, ScienceDirect, PubMed, and Scopus databases. The terms or combination of terms used for the search include nursing, immunotherapy, cancer, cancer treatment, nursing practice, oncology; oncology nursing, targeted therapy; and adverse events from 2004 to December 2023. The articles that satisfied the inclusion criteria were fully reviewed and references from the selected literature have been cited where appropriate.

## 3 Targeted therapy

**Fig. 1:**
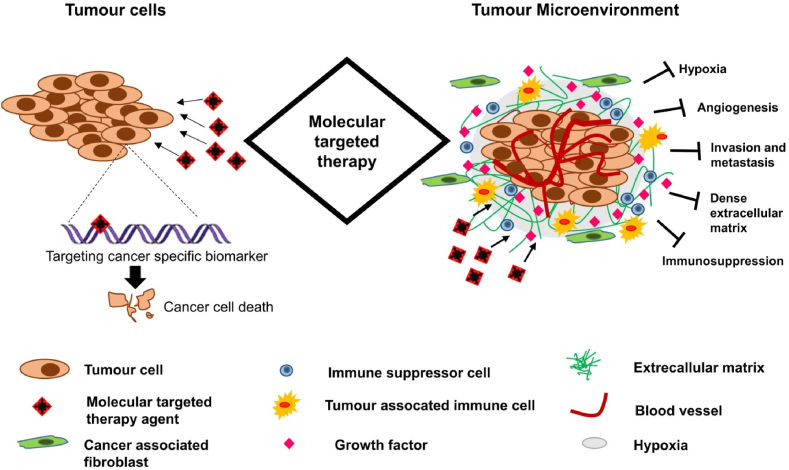
The general mechanism of action of molecular targeted therapy. Targeted therapies primarily focus on targeting certain cancer-linked molecules that are greatly expressed in cancer cells or via controlling the tumor microenvironment related to hypoxia, metastasis or tumor vasculature. Reproduced with permission from Elsevier, Reference [14].

Targeted therapies have the capacity to directly act on cancerous cells via suppressing cell migration, differentiation, and proliferation [10]. The composition of the tumor microenvironment includes immune cells and local blood vessels, which may be changed by targeted drugs to hinder tumor growth and ratify powerful immune surveillance and attack (Fig. 1). In targeted therapies, small molecules including monoclonal antibodies (MoAbs) play significant roles [[11], [12], [13]]. It has been observed that small molecules with molecular weights less than 900 Da may penetrate into cells and then work within cells to deactivate certain enzymes, which further interferes with tumor cell growth and eventually induce apoptosis. Most of the molecular targets involve proteasomes, poly ADP-ribose polymerase, and cyclin-dependent kinases. For example, ribociclib for metastatic breast cancer, rucaparib for BRCA-positive ovarian cancer, and carfilzomib for multiple myeloma [14]. On the other hand, for targets outside cells including therapeutic antibodies or MoAbs, membrane-bound sites or cell surface receptors can detect and bind with them to directly control downstream cell death and cell cycle progression. Moreover, specific MoAbs act on cells instead of cancerous cells including immune cells, which can further aid the manipulation of the immune system to attack human cancer [15].

Three major types of targeted therapies include MoAbs, small molecule tyrosine kinase inhibitors (TKIs), and vaccines. It has been observed that vaccines do not work particularly on signaling pathways in cancer cells, however mostly play a role to cause activation of the immune system of the body to detect and attack cancer cells. It is now well known that small molecules can effectively cross cell membranes. The suffix “nib" is present in small molecule TKIs, for example sorafenib, sunitinib, pazopanib, imatinib, and erlotinib. Since small-molecule drugs are administered orally, therefore food-drug and drug-drug interactions need to be considered. Most of the patients obtain their treatment at home, therefore oncology nurses adopt approaches to ensure adherence to time and dose requirements as well as management of side effects. MoAbs act outside of the cell to prevent receptors and signaling molecules from interacting. Furthermore, MoAbs have the capacity to transport toxins or radioactive molecules to cancer cells. Once within the cells, the cytotoxin is secreted which results in cell death. MoAbs have the capacity to play roles outside the cells to induce immune responses and eliminate cancer cells. In addition, MoAbs are derived from human, chimeric, or mouse antibodies and are intravenously administered [4]. Interestingly, MoAbs derived from human antibodies are more tolerable and generate fewer infusion reactions. Examples of such antibodies include bevacizumab, panitumumab, trastuzumab, rituximab, and tositumomab [16].

In general, the suffix "-mab" is used to denote monoclonal antibodies. Mo before mab is used to denote mouse-derived MoAbs, for example tositumomab. Xi before mab is used to indicate a cross or chimeric between a human and a mouse, for example rituximab. On the other hand, Zu before mab indicates humanized antibodies (for example bevacizumab or trastuzumab), while mu before mab denotes fully human-derived antibodies, for example panitumumab. Indeed, fewer infusion reactions can be anticipated if the antibodies are more humanized. Tu is used prior to the type of antibody to indicate that the tumor is the target, for example panitumumab or trastuzumab, Ci denotes a circulatory target (for example bevacizumab), and Li is used to denote that the MoAb is an immunomodulatory, for example ipilimumab. In order to have better command regarding cancer therapies, oncology nurses need to understand the naming conventions, routes of administration, and management of side effects [17]. Current targeted therapies for cancer have been summarized in Table 1. The nurses are required to have good knowledge regarding the important and common adverse effects to intervene and educate the patients about the self-care measures since some of the adverse effects could be dangerous.

**Table 1:** Currently approved targeted therapy drugs for cancer.

Class/Drug (Generic name)	Cancer type	Common adverse reactions	References
Anaplastic lymphoma kinase (ALK) inhibitors			
Alectinib	ALK-positive metastatic non-small cell lung cancer (mNSCLC)	Myalgia, constipation, edema, fatigue	[[18], [19], [20]]
Ceritinib	ALK-positive mNSCLC	Weight loss, decreased appetite, abdominal pain, vomiting, nausea, diarrhea, fatigue	[21]
Crizotinib	ALK-positive locally advanced or metastatic NSCLC	Dizziness, upper respiratory infection, fatigue, edema, neuropathy, decreased appetite, elevated transaminases, diarrhea, vomiting, constipation, nausea, vision disorders	[22,23]
Brigatinib	ALK-positive mNSCLC	Headache, cough, nausea, fatigue, diarrhea	[20,24]
**Angiogenesis inhibitors**			
Axitinib	Advanced renal cell carcinoma (aRCC)	Constipation, asthenia, vomiting, weight loss, Palmar-plantar erythrodysesthesia syndrome (PPES), dysphonia, nausea, decreased appetite, fatigue, hypertension (HTN), diarrhea	[25,26]
Cabozantinib	aRCC; hepatocellular carcinoma; differentiated thyroid cancer (DTC)	Constipation, weight loss, vomiting, HTN, PPES, decreased appetite, nausea, fatigue, diarrhea, hypophosphatemia, hypocalcemia, thrombocytopenia, neutropenia, elevated alkaline phosphatase, abdominal pain, dysgeusia, hair color alterations, stomatitis	[[27], [28], [29], [30]]
Pazopanib	aRCC; advanced soft tissue sarcoma (STS)	Anorexia, nausea, vomiting, hair color changes, HTN, diarrhea	[31]
Levatinib	Locally recurrent or progressive; metastatic, radioactive iodine-refractory DTC	Weight loss, abdominal pain, proteinuria, headache, nausea, diarrhea, HTN, PPES, decreased appetite, arthralgia/myalgia, dysphonia, stomatitis, vomiting, fatigue	[32,33]
Regorafenib	Metastatic colorectal cancer (mCRC)	Nausea, weight loss, fever, HTN, rash, mucositis, decreased appetite/food intake, hyperbilirubinemia, dysphonia, asthenia/fatigue, infection, diarrhea, hand foot skin reaction (HFSR), pain	[3,31]
Sunitinib	Gastrointestinal stromal tumor (GIST) after disease progression on or intolerance to imatinib mesylate; aRCC; progressive, well-differentiated pancreatic neuroendocrine tumors (PNET)	Anorexia, cough, bleeding, dyspnea, arthralgia, extremity pain, backpain, headache, altered taste, hair color changes, dry skin, HFSR, rash, skin discoloration, HTN, dyspepsia, peripheral edema, abdominal pain, nausea, constipation, mucositis/stomatitis, fatigue, vomiting, fever, asthenia, diarrhea	[3,34]
Sorafenib	Locally recurrent or metastatic, progressive, differentiated thyroid carcinoma refractory to radioactive iodine treatment; unresectable hepatocellular carcinoma; aRCC	Hemorrhage, HTN, gastrointestinal and abdominal pain, nausea, weight loss, decreased appetite, rash, HFSR, infection, fatigue, diarrhea, alopecia	[35,36]
Ziv-aflibercept	mCRC	Abdominal pain, dysphonia, decreased appetite, headache, increased serum creatinine, weight loss, epistaxis, fatigue, thrombocytopenia, stomatitis, HTN, proteinuria, neutropenia, diarrhea, leukopenia, elevated levels of aspartate aminotransferase (AST) and alanine aminotransferase (ALT)	[37,38]
Vandetanib	Symptomatic or progressive medullary thyroid cancer in patients with unresectable locally advanced or metastatic disease	Abdominal pain, decreased appetite, HTN, headache, diarrhea/colitis, nausea, aneiform dermatitis, rash,	[39,40]
**Bcl-2 inhibitor**			
Venetoclax	Acute myeloid leukemia (AML); small lymphocytic lymphoma (SLL); chronic lymphocytic leukemia (CLL);	Fatigue, thrombocytopenia, upper respiratory infection (URI), anemia, nausea, diarrhea, neutropenia	[3,41]
**BCR-ABL kinase inhibitors**			
Bosutinib	Philadelphia chromosome-positive (Ph+) chronic myelogenous leukemia (CML)	Headache, fatigue, cough, pyrexia, anemia, abdominal pain, nausea, respiratory tract infections, thrombocytopenia, vomiting, rash, diarrhea	[3]
Imatinib mesylate	Ph + CML, Ph + acute lymphoblastic leukemia (ALL)	Fatigue, diarrhea, nausea, muscle cramps, edema, abdominal pain, vomiting, rash, musculoskeletal pain	[42,43]
Dasatinib	Ph + CML	Musculoskeletal pain, nausea, fatigue, dyspnea, hemorrhage, skin rash, headache, diarrhea, fluid retention events, myelosuppression	[42,44]
Ponatinib	Chronic phase, accelerated phase, or blast phase CML; Ph + ALL	Increased level of serum lipase, pyrexia, fatigue, arthralgia, nausea, headache, vomiting, constipation, diarrhea, rash, myalgia, extremity pain, abdominal pain, HTN, dry skin	[45]
Nilotinib	Ph + CML	Arthralgia, cough, vomiting, night sweats, constipation, diarrhea, nausea, nasopharyngitis, fatigue, headache, myelosuppression, pruritus, pyrexia, rash	[46]
**MEK inhibitors**			
Trametinib	Unresectable or metastatic melanoma with BRAF V600E or V600K mutation	HTN, hemorrhage, decreased appetite, dry skin, pyrexia, peripheral edema, vomiting, diarrhea, chills, rash, nausea, lymphedema, diarrhea, rash	[47,48]
Cobimetinib	Unresectable or metastatic melanoma with BRAF V600E or V600K mutation	Increased level of creatine phosphokinase, vomiting, pyrexia, hypophosphatemia, lymphopenia, hyponatremia, nausea, photosensitivity reaction, diarrhea	[49,50]
**BRAF inhibitors**			
Dabrafenib	Unresectable or metastatic melanoma with BRAF V600E or V600K mutations	Fatigue, hemorrhage, edema, decreased appetite, dry skin, PPES, diarrhea, vomiting, nausea, decreased appetite, alopecia, papilloma, arthralgia, pyrexia, headache, hyperkeratosis	[51]
Vemurafenib	Unresectable or metastatic melanoma with BRAF V600E mutation	Prolonged QT-interval, skin papilloma, nausea, alopecia, pruritus, fatigue, photosensitivity reaction, rash, arthralgia	[52,53]
**Bruton's tyrosine kinase (BTK) inhibitors**			
Acalabrutinib	Adult patients with mantle cell lymphoma (MCL)	Fatigue, bruising, thrombocytopenia, myalgia, diarrhea, neutropenia, headache, anemia	[54,55]
Ibrutinib	MCL; CLL; SLL; Waldenström's macroglobulinemia; marginal zone lymphoma	hemorrhage, muscle spasms, diarrhea, pyrexia, fatigue, neutropenia, bruising, nausea, rash, musculoskeletal pain, anemia, thrombocytopenia	[[56], [57], [58]]
**Cyclin-dependent kinase inhibitors**			
Abemaciclib	Hormone receptor (HR)-positive, human epidermal growth factor receptor 2 (HER2)-negative advanced or metastatic breast cancer	Anemia, thrombocytopenia, headache, decreased appetite, abdominal pain, infections, vomiting, leukopenia, fatigue, nausea, neutropenia, diarrhea	[[59], [60], [61]]
Ribociclib	HR-positive, HER2-negative advanced or metastatic breast cancer	Alopecia, back pain, diarrhea, headache, constipation, vomiting, leukopenia, fatigue, nausea, neutropenia	[62,63]
Palbociclib	HER2-negative advanced or metastatic breast cancer	Thrombocytopenia, asthenia, nausea, pyrexia, decreased appetite, vomiting, rash, alopecia, infections, diarrhea, anemia, stomatitis, fatigue, leukopenia, neutropenia	[64]
**Small-molecule epidermal growth factor receptor (EGFR) tyrosine kinase inhibitors (TKIs)**			
Afatinib	mNSCLC with EGFR mutations	Stomatitis, pruritus, vomiting, nausea, decreased appetite, dry skin, paronychia, dermatitis, rash/acneiform, diarrhea	[65,66]
Gefitinib	mNSCLC with tumors showing EGFR exon 19 deletions or exon 21 (L858R) substitution mutations	Diarrhea, skin reactions	[67,68]
Erlotinib	mNSCLC with tumors showing EGFR exon 19 deletions or exon 21 (L858R) substitution mutations	Anorexia, vomiting, nausea, cough, dyspnea, fatigue, diarrhea, rash	[67,68]
Neratinib	Early stage HER2-overexpressed/amplified breast cancer	Dry skin, urinary tract infection (UTI), weight loss, abdominal distention, nail disorder, dyspepsia, muscle spasms, decreased appetite, stomatitis, rash, vomiting, fatigue, abdominal pain, nausea, diarrhea	[69]
Lapatinib	Advanced or metastatic breast cancer whose tumors overexpress HER2; HR-positive metastatic breast cancer that overexpresses the HER2 receptor	Nausea, rash, fatigue, diarrhea, vomiting, PPES	[70,71]
Osimertinib	Metastatic EGFR T790 M mutation-positive NSCLC	dry skin, fatigue, nail toxicity, rash, diarrhea	[72,73]
**FLT3 kinase inhibitor**			
Midostaurin	Newly diagnosed FLT3 mutation-positive AML; aggressive systemic mastocytosis (ASM), systemic mastocytosis with associated hematological neoplasm (SM-AHN), or mast cell leukemia	Hyperglycemia, vomiting, dyspnea, headache, pyrexia, constipation, fatigue, abdominal pain, edema, diarrhea, epistaxis, musculoskeletal pain, petechiae, URI, headache, mucositis, nausea, febrile neutropenia	[74,75]
**Hedgehog pathway inhibitors**			
Sonidegib	Locally advanced basal cell carcinoma (BCC)	Vomiting, pruritus, musculoskeletal pain, myalgia, pain, headache, decreased appetite and weight, diarrhea, nausea, fatigue, dysgeusia, alopecia, muscle spasms	[76]
Vismodegib	Metastatic BCC; locally advanced BCC	Weight loss, decreased appetite, ageusia, vomiting, arthralgias, constipation, diarrhea, nausea, fatigue, dysgeusia, alopecia, muscle spasms	[[77], [78], [79]]
**Histone deacetylase (HDAC) inhibitors**			
Belinostat	Relapsed or refractory peripheral T-cell lymphoma (PTCL)	Pyrexia, vomiting, anemia, fatigue, nausea,	[80,81]
Romidepsin	Cutaneous T-cell lymphoma (CTCL)	Anemia, anorexia, vomiting, fatigue, nausea, thrombocytopenia, neutropenia, infections, lymphopenia	[82,83]
Panobinostat	Multiple myeloma	Pyrexia, vomiting, fatigue, nausea, decreased appetite, peripheral edema, diarrhea	[84,85]
Vorinostat	Progressive, persistent, or recurrent CTCLs	Anorexia, dysgeusia, nausea, thrombocytopenia, fatigue, diarrhea	[86]
**Isocitrate dehydrogenase-2 (IDH2) inhibitor**			
Enasidenib	Relapsed or refractory AML with an IDH2 mutation	Diarrhea, decreased appetite, vomiting, nausea	[87,88]
**Mammalian target of rapamycin (mTOR) inhibitor**			
Temsirolimus	aRCC	Anorexia, edema, nausea, mucositis, asthenia, rash	[89,90]
Everolimus	Advanced HR-positive, HER2-negative breast cancer; adults with progressive unresectable PNET; aRCC; adults with renal angiomyolipoma	Cough, decreased appetite, headache, nausea, abdominal pain, diarrhea, stomatitis, fever, asthenia, edema, fatigue, rash	[91]
**Poly (ADP-ribose) polymerase (PARP) inhibitors**			
Olaparib	Deleterious or suspected deleterious germline BRCA-mutated (gBRCAm) advanced ovarian cancer; HER2-negative metastatic breast cancer; recurrent epithelial ovarian, fallopian tube or primary peritoneal cancer	Stomatitis, constipation, decreased appetite, dyspepsia, headache, dysgeusia, arthralgia/myalgia, diarrhea, URI/influenza, nasopharyngitis, vomiting, fatigue, nausea, anemia	[92,93]
Niraparib	Recurrent epithelial ovarian, fallopian tube, or primary peritoneal cancer who are in a partial or complete response to platinum-based chemotherapy	Anxiety, back pain, dyspepsia, HTN, rash, cough, dyspnea, nasopharyngitis, insomnia, dysgeusia, dizziness, headache, arthralgia, myalgia, UTI, decreased appetite, fatigue/asthenia, dry mouth, diarrhea, mucositis/stomatitis, abdominal pain/distention, vomiting, constipation, nausea, palpitations, leukopenia, neutropenia, anemia, thrombocytopenia	[94,95]
Rucaparib	Recurrent epithelial ovarian, fallopian tube, or primary peritoneal cancer who are in a partial or complete response to platinum-based chemotherapy; deleterious BRCA mutation (germline and/or somatic)-associated epithelial ovarian, fallopian tube, or primary peritoneal cancer who have been treated with two or more chemotherapies	Dyspnea, thrombocytopenia, diarrhea, decreased appetite, constipation, dysgeusia, abdominal pain, anemia, vomiting, fatigue, nausea	[96,97]
**Phosphatidylinositol 3-kinase (PI3K) inhibitor**			
Copanlisib	Relapsed follicular lymphoma (FL) (adult patients)	Thrombocytopenia, lower respiratory infection, nausea, neutropenia, leukopenia, HTN, reduced general strength and energy, diarrhea, hyperglycemia	[98,99]
Idelalisib	Relapsed follicular B-cell non-Hodgkin lymphoma; relapsed SLL; relapsed CLL, in combination with rituximab;	Rash, pneumonia, abdominal pain, pyrexia, cough, nausea, fatigue, diarrhea	[100,101]
**Proteasome inhibitors**			
Bortezomib	Mantle cell lymphoma; multiple myeloma	Constipation, anorexia, pyrexia, rash, lymphopenia, vomiting, leukopenia, anemia, neuralgia, fatigue, peripheral neuropathy, neutropenia, thrombocytopenia, diarrhea, nausea	[102,103]
Ixazomib	In combination with dexamethasone and lenalidomide for the treatment of patients with multiple myeloma	Back pain, nausea, vomiting, peripheral neuropathy, constipation, peripheral edema, thrombocytopenia, diarrhea	[104,105]
Carfilzomib	Multiple myeloma	Cough, diarrhea, pyrexia, edema, headache, dyspnea, nausea, thrombocytopenia, fatigue, anemia	[106,107]

### 3.1 Nursing assessment and management of adverse events in cancer treatment with targeted therapies

In general, all the drugs that belong to the same class share a common mechanism of action, therefore some of the adverse effects are predictable. With the proper knowledge regarding the class effects, drugs and their mechanisms of action, and predicted adverse effects, the nurses can evaluate the patients for possible toxicity, collaborate with practitioners/physicians and intervene, as well as can provide correct patient education in self-care. Indeed, nurses need to have a better understanding regarding each drug prior to administering it, since numerous drugs sound alike to recognize drug-specific nursing care management approaches. The nurses should have the capacity to evaluate hypersensitivity reactions and intervene in an emergency. Furthermore, all symptoms and signs are not the same. In this regard, for instance the nurses need to know that erlotinib (an EGFR inhibitor) associated diarrhea is very different from nivolumab (a PD-1 inhibitor) associated diarrhea, which might be symptomatic of immune-mediated colitis and might be life-threatening [108]. The nursing care plan should be updated and evidence-based [109]. Immunotherapies are being developed rapidly and guidelines are still being written [109]. Cytokine release syndrome associated with CAR-T cell therapy might be fatal and severe, and nurses need to have a better understanding regarding the treatment implications. Most of the drugs have the potential to cause embryo-fetal developmental toxicity, therefore it is crucial to educate female patients of child-bearing age to utilize very effective contraception. Moreover, since the costs of most of these therapies are important, therefore nurses should assist the patients or involve others in findings resources to help the patients to receive these therapies [109].

## 4 Immunotherapy

**Fig. 2:**
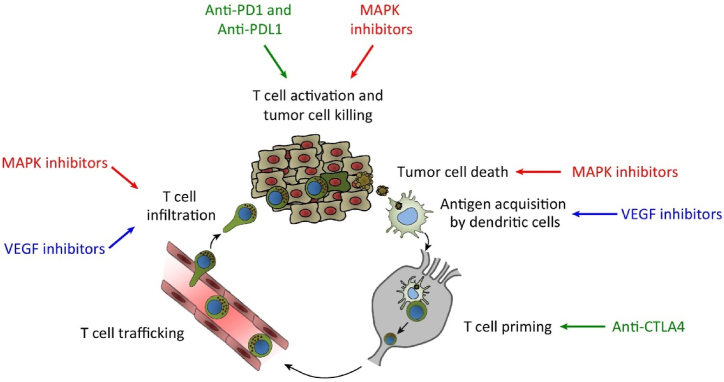
The mode of action of various immunotherapies. Reproduced with permission from Elsevier, Reference [113].

Immunotherapy involves the usage of materials that enhance and/or reestablish the ability of the immune system to avert and fight diseases [110]. The aim of immunotherapy is to balance the immune system to eradicate cancer cells (Fig. 2), while not generating unchecked autoimmune inflammatory reactions that can cause therapeutic restrictions of immunotherapies [111]. The innate immune responses are restricted to the secretion of cytokines that involve immune cells to instigate non-specific immune responses. On the other hand, adaptive immune responses have a significant contribution in the immune response to cancer cells owing to their capacity to precisely target non-self antigens [110,111]. In the tumor microenvironment, cancer-specific antigens generated during oncogenesis are processed and captured via dendritic cells (DCs) [111]. These DCs are activated and move to tumor-draining lymph nodes, after additional pro-inflammatory signals, where they induce the activation and also differentiation of naïve T cells to become effector T cells that have the capacity to kill cancer cells. Furthermore, activated effector T cells move from the lymph nodes via blood vessels to the tumor and infiltrate into the tumor bed via a multistep procedure that includes preliminary adhesive interactions between the vascular endothelial cells and T cells, after that transendothelial migration into the tumor [112,108]. After the tumor bed infiltration by T cells, identification of certain tumor antigens results in T cell killing of cancer cells, which can further induce additional antigen secretion and successive stimulation of extra rounds of the cancer-immunity cycle (Fig. 2) [109].

Immune therapies include MoAbs, oncolytic virus therapy, cancer vaccines, cytokine drugs, checkpoint inhibitors, and chimeric antigen receptor (CAR) T-cell therapy. Immunotherapies aim to elevate immune activity and they are classified based on their mechanisms of action. In addition, immunotherapies can be grouped into passive and active immunotherapies. Active immunotherapies directly induce immune responses, lasting responses, and immune memory [114]. Oncolytic vaccines are examples of active immunotherapies. Passive immunotherapies (for example MoAbs) can generate precise but often short-lived responses; therefore these agents need to be regularly administered. Moreover, there can be an interruption in radiologic and clinical response to immunotherapies. This can take place owing to the time requires for an immune response to take place and then the time requires for the T cells to eliminate the tumor. There are several advantages of using immune therapy owing to their immune memory properties including prolonged immune action following termination of therapy, which might result in sustained antitumor activities and long-term overall survival [114]. However, some reports indicate that tumors that primarily respond to immune therapy might eventually become resistant. Multiple studies are ongoing to evaluate the effects of therapies to fight against immunotherapy resistance [6,115]. Approved immunotherapies that are approved for cancer treatment have been summarized in Table 2.

**Table 2:** Approved immunotherapy drugs for cancer treatment.

Category/Drug	Target	Indications	Treatment-related adverse events	References
Checkpoint inhibitors				
Nivolumab	PD-1	Renal cell carcinoma (RCC), non-small cell lung cancer (NSCLC), urothelial carcinoma, small cell lung cancer (SCLC), head and neck squamous cell cancer (HNSCC), pleural mesothelioma, colorectal cancer (CRC), microsatellite instability-high (MSI-H) or mismatch repair deficient (dMMR), hepatocellular carcinoma (HCC), classical hodgkin lymphoma, esophageal squamous cell carcinoma (ESCC), melanoma	Hypersensitivity, erythema, renal failure, muscle spasms, peripheral neuropathy, elevated amylase levels, infusion-related reaction, thyroiditis, hepatitis, and maculopapular rash	[[114], [116], [117]]
Ipilimumab	CTLA-4	Metastatic melanoma	Nephritis, hypophysitis, dermatitis, hepatitis, pneumonitis, and enterocolitis	[118,113]
Pembrolizumab	PD-1	Melanoma, NSCLC, HNSCC, urothelial carcinoma, primary mediastinal B-cell lymphoma (PMBCL), endometrial carcinoma, classical hodgkin lymphoma (cHL)	Pruritus, rash, less frequently vitiligo	[116,115]
Atezolizumab	PD-1	NSCLC, urothelial carcinoma	Rash, hepatitis (laboratory abnormalities), and hypothyroidism	[119,120]
Durvalumab	PD-1	Urothelial carcinoma	Dermatitis, pneumonitis, thyroiditis, and hepatitis	[119,121]
Avelumab	PD-1	Urothelial carcinoma, Merkel-cell carcinoma	Urticaria, abdominal pain, back pain, wheezing, dyspnea, hypertension, flushing, chills, pyrexia	[119,122]
**Chimeric antigen receptor (CAR) T-cell therapy**				
Axicabtagene ciloleucel	CD19	Large B-cell lymphoma (LBCL)	Anxiety, insomnia, delirium, aphasia, dizziness, tremor, headache, and encephalopathy	[123]
Idecabtagene vicleucel	BCMA	Relapsed or refractory multiple myeloma (r/r MM) after four or more prior lines of therapy	Chimeric Antigen Receptor T-cell associated hemophagocytic lymphohistiocytosis, immune effector cell-associated neurotoxicity syndrome, and cytokine release syndrome	[123,124]
Brexucabtagene autoleucel	CD19	Relapsed/refractory mantle cell lymphoma (r/r MCL)	Encephalopathy, persistent cytopenia, hypogammaglobulinaemia, hypophosphataemia and hypokalaemia	[123]
Lisocabtagene maraleucel	CD19	r/r Large B-cell lymphoma (LBCL) after two or more lines of systemic therapy	Cytokine release syndrome, fatigue, neutropenia, and anemia	[123]
Tisagenlecleucel	CD19	Relapsed or refractory follicular lymphoma after two or more lines of therapy	Headache, febrile neutropenia, decreased appetite, pyrexia, and cytokine release syndrome	[123]
**Cytokine drugs**				
Interferon alfa-2b	Interferon alpha and beta receptor subunit 1; interferon alpha and beta receptor subunit 2	Malignant melanoma, follicular lymphoma	Insomnia, reduced appetite, irritability, fatigue	[125,126]
Aldesleukin	Interleukin-2 (IL-2) receptor	Metastatic RCC, metastatic melanoma	Pruritus, nausea, and fatigue	[127,128]
**Cancer vaccines**				
Bacillus Calmette–Guérin (BCG)	Bladder cancer cells	Early-stage bladder cancer	General malaise, fatigue, and pyrexia	
Provenge	Prostatic acid phosphatase	Prostate cancer	Arthralgia, nausea, back pain, fever, fatigue, and chills	[129]
**Oncolytic virus therapy**				
Talimogene Laherparepvec (T-VEC)	Melanoma cells	Metastatic melanoma	Influenza-like illness, nausea, pyrexia, chills, and fatigue	[130]
**Monoclonal antibodies (MoAbs)**				
Cetuximab	EGRF, HER-1	Colorectal carcinoma, head and neck cancer	Fatigue, nausea, decreased appetite, acneiform dermatitis, diarrhea, reduced hemoglobin, and abnormal creatinine levels	[131]
Ibritomomab tiuxitan	CD20	Non-Hodgkin's lymphoma	Headache, fever, vomiting, chills, nausea, and asthenia	[132]
Alemtuzumab	CD52	Chronic lymphocytic leukemia (CLL)	Immune thrombocytopenia, nephropathies, thyroid autoimmune disease	[133]
Pertuzumab	EGFR2, HER-2	Breast carcinoma	left ventricular systolic dysfunction, neutropenia, diarrhea, and cardiotoxicity	[134]
Trastuzumab	HER-2	Breast cancer	Nausea, pain, fever, asthenia, and chills	[135]
Ipilimumab	CTLA-4	Melanoma	hepatic, endocrine, gastrointestinal, and dermatologic events	[136]
Panitumumab	EGRF, HER-1	Colorectal carcinoma	Hypomagnesemia, pruritus, erythema, and dermatitis acneiform	[137]
Bevacizumab	VEGF	Colorectal carcinoma, glioblastoma multiforme, RCC, lung carcinoma, cervical carcinoma	Fatigue, neuropathy, abdominal pain, thrombocytopenia, hypertension, and bleeding	[138]
Rituximab	CD20	CLL, non-Hodgkin lymphoma (NHL)	Rash, pruritus, nausea, weakness, headache, chills, and fever	[139]
Ofatumumab	CD20	CLL	upper respiratory tract infection, urinary tract infection, nasopharyngitis, injection-site reaction, and headache	[140]
Tositumomab	CD20	NHL	Vomiting, nausea, infection, chills, fever, headache, and transient marrow suppression,	[141]

FDA approval of immune checkpoint inhibitors (ICIs) has significantly transformed the cancer treatment strategy for multiple diseases that were once thought incurable. Now, individuals with renal cell carcinoma (RCC), metastatic melanoma, and lung cancer have hope and active therapies are playing role in extending their life. Another important discovery in cancer treatment is chimeric antigen-receptor T-cell (CAR T-cell) therapies. In the treatment of certain hematologic malignancies, two different CAR T-cell therapies including axicabtagene ciloleucel and tisagenlecleucel were approved [142]. Other important immunotherapies include modified viral targets and oncolytic vaccines. A number of vaccine candidates were found effective in animal studies, however their effectiveness was not confirmed in clinical studies. The reason for the failure of cancer vaccines in the metastatic setting was mainly because of the immune systems of patients being in a state of chronic inflammation, which further results in T-cell exhaustion [143]. However, studies are ongoing in exploring the usage of vaccines in cancer recurrence following complete surgical resection. Another promising immunotherapy is modified viral targets. Talimogene laherparepvec (T-VEC) was the first FDA-approved oncolytic virus therapy. T-VEC contains a genetically engineered herpes virus (an oncolytic herpes virus) that is used in the treatment of surgically unresectable melanoma [144,145].

Still, cytotoxic chemotherapy is considered as the first line of treatment for advanced-stage head and neck cancers. Moreover, second-line therapeutic options were also limited, up until the arrival of ICIs. In the treatment of head and neck cancers, nivolumab was the first immunotherapy approved by FDA based on the findings obtained from a clinical trial [146], while pembrolizumab obtained FDA approval as second-line therapy based on the findings obtained from another clinical trial [147]. Esophageal cancer can be treated, however it is rarely curable in metastatic or advanced disease. At 5 years, survival rates for advanced stages esophageal cancer are usually 5–20 %. Results obtained from a clinical trial confirmed the ability of pembrolizumab in improving the overall survival rate in patients with programmed death ligand-1 (PD-L1) and PD-L1 combined positive score (CPS) > 10 [148]. This CPS was established to assess the number of PD-L1 staining cells as compared to all viable tumor cells, and it has become a surrogate marker for individuals who might benefit from pembrolizumab treatment. In addition, it has been confirmed by a clinical trial that the addition of pembrolizumab to chemotherapy and trastuzumab markedly ameliorated objective response rate, induced complete responses in some participants, and significantly decreased the tumor size in case of unresectable or metastatic, HER2-positive gastric or gastro-esophageal junction adenocarcinoma [149].

As mentioned before, ipilimumab (anti-CTLA-4 inhibitor) is the first FDA-approved ICI that is used to treat metastatic melanoma [150]. After ipilimumab, several anti-PD-1/PD-L1 inhibitors have been developed that have been approved by FDA in the treatment of several diseases [[151], [152], [153], [154], [155], [156], [157], [158]]. Following the success of therapy with single-agent ICI resulted in dual combination ICI therapy has been introduced to ameliorate therapeutic outcomes. Ipilimumab/nivolumab obtained its first approval as dual ICI therapy to treat metastatic melanoma [[159], [160], [161], [162], [163]]. Owing to the success of treatment with ICI in metastatic settings, various studies are now evaluating their effects in adjuvant settings. Former studies have compared ipilimumab versus placebo in high-risk (stage III) resected melanoma patients. Because of the improvement observed in the case of overall survival and relapse-free survival resulted in the approval of ipilimumab as adjuvant therapy [164]. Pembrolizumab and nivolumab also have been approved as adjuvant therapy in case of melanoma [165,166]. Unfortunately, this achievement came with the consequences of elevated levels of autoimmune toxicity. Nonetheless, researchers are currently studying to explore approaches to control the immune system of the body to maximize efficacy and regulate the often severe and unpredictable immune-related adverse events (irAEs) associated with therapies. Furthermore, researchers are still exploring the potential of ICIs in combination with chemotherapy and other targeted therapies [167].

### 4.1 Nursing assessment and management of adverse events in cancer treatment with immunotherapies

Indeed, immunotherapies have changed the paradigm of cancer treatment with lesser side effects and better outcomes. However, adverse events are observed along with their use. Because of the elevated level of immune system stimulation, the normal homeostatic control mechanisms providing protection to the body from its own immune response can become disturbed, which can further result in various side effects known as irAEs. It has been reported that irAEs can play role in morbidity and in numerous cases can result in the discontinuation of therapies with unpredictable effects on the course of the disease of patients. Oncology nurses ought to have a good understanding regarding the mechanisms and adverse events linked with such therapies. Nurses play a significant role in the identification of irAEs. Furthermore, irAEs might include several systems and therefore it is important to detect and manage these adverse events as soon as possible. Different irAEs and the recommended management strategies along with the extent of toxicity have been summarized in Table 3.

**Table 3:** Management of immune-related adverse effects.

Common adverse reactions	Degree of toxicity	Management of immune-related adverse effects	Reference
Colitis	Grade 1	Antidiarrheal agents, oral fluids, continue immunotherapy	[168]
	Grade 2	A nephrology consultation, oral prednisone 0.5–1 mg/kg/day, pause immunotherapy	
	Grade 3	Oral prednisone 1–2 mg/kg/day, stop immunotherapy	
	Grade 4	Start dialysis, IV methylprednisone 1–2 mg/kg/day	
Hepatitis	Grade 1	Check hepatotoxic drug, continue immunotherapy	[168]
	Grade 2	Pause immunotherapy, oral prednisone 0.5–1 mg/kg/day	
	Grades 3-4	A hepatology consultation, do not offer infliximab, immunomodulatory therapy, IV methylprednisone 1–2 mg/kg/day, stop immunotherapy	
Pneumonitis	Grade 1	Pause immunotherapy	[168]
	Grade 2	Bronchoscopy and/or bronchoalveolar lavage, empirical antibiotics, oral prednisone 1–2 mg/kg/day, pause immunotherapy	
	Grades 3–4	IV methylprednisone 1–2 mg/kg/day, stop immunotherapy, empirical antibiotics, additional immunomodulatory therapy, a pulmonology consultation, intravenous immunoglobulin if there is no improvement	
Skin rash	Grade 1	Continue immunotherapy, oral antihistamines for pruritus, topical emollients, topical corticosteroids	[168]
	Grade 2	Immunotherapy can be paused, oral prednisone 1 mg/kg/day,	
	Grade 3	A dermatology consultation, pause immunotherapy, oral prednisone 1–2 mg/kg/day	
	Grade 4	Immunomodulatory therapy in steroid non-responders, IV prednisone 1–2 mg/kg/day	
Inflammatory arthritis	Grade 1	Non-steroidal anti-inflammatory drugs or acetaminophen, continue immunotherapy	[168]
	Grade 2	A rheumatology consultation, - Intra-articular steroid injection, oral prednisone 10–20 mg/day, immunotherapy can be paused	
	Grades 3–4	Pause immunotherapy, immunomodulatory therapy in steroid non-responders, oral prednisone 0.5–1 mg/kg/day	
Hypophysitis	Grade 1	Immunotherapy can be paused, glucocorticoid replacement with stress day rules	[168]
	Grade 2	Oral prednisone 0.5–1 mg/kg/day, an endocrinology consultation	
	Grades 3–4	Oral prednisone 1–2 mg/kg/day	

## 5 Combinations of immunotherapies and targeted therapies in cancer treatment

Cancer therapy involving the combinations of immunotherapies and targeted therapies can play synergistic roles in cancer treatment. Moreover, targeted therapies can rapidly induce tumor regressions and thus can reduce tumor-related immunosuppression, which can further facilitate immunotherapies to exert more stronger cytotoxicity [169]. Success has been obtained in cancer treatment by using monotherapy, however there are major limitations in terms of the duration of therapy and response rates [170]. Because of these limitations and promising preclinical evidence regarding the effectiveness of combinations of immunotherapies with other cancer therapies, there is a growing interest in the combinations of immunotherapies and targeted therapies in cancer treatment [171]. Nonetheless, the rational design of these combined approaches needs an improved understanding of the effects of each anticancer therapy on host antitumor immunity [172]. Various preclinical studies have already confirmed the combined effects of immunotherapy and histone deacetylase (HDAC) inhibitors. These enhanced combinatorial activities were facilitated via improved antigen presentation by tumor cells and elevated activity of immune cells, both resulted in an elevated level of antitumor effect [173].

Furthermore, HDAC inhibitors were combined with various immune-activating antibodies including anti-CD40 and anti-CD137, which treated previously established subcutaneous tumors [174]. In this model, the mechanism was found to be reliant on HDAC inhibitor therapy-mediated tumor cell apoptosis, which induced antigen-cross-presentation to increase the survival and proliferation of CD8^+^ cytotoxic T lymphocytes [174]. In combination with immunotherapy, the potentially useful actions of HDAC inhibitors need to be balanced with the results that confirm the immune suppressive functions of HDAC inhibitors [[175], [176], [177]], particularly via elevating the inhibitory functions of T regulatory cells [178,179]. In lymphocytes, HDAC inhibitors are also strong suppressors of cell cycle proliferation [177]. Thus, intense mechanistic studies are needed if such combinations are evaluated in the clinic [180]. Specific pharmacologic effects of type I RAF inhibitors might further affect the capacity of targeted therapies to interact with the immune systems. It has been observed that RAF inhibitors induced paradoxical effects in wild-type BRAF cells, which further activated mitogen-activated protein kinase (MAPK) signaling pathway. This mechanism depends on the CRAF transactivation via a partly blocked wild-type CRAF:BRAF dimer, especially when the upstream activating signals are strong [[181], [182], [183]]. Interestingly, this contradictory activation of MAPK is the foundation for the discovery of cutaneous squamous cell carcinoma and various other RAS-mediated secondary cancers in individuals receiving BRAF inhibitors [[184], [185], [186]]. T cells are wild-type for BRAF and their induction of TCR causes activation of the MAPK signaling pathway, therefore BRAF inhibitors might directly cause activation of lymphocytes via contradictory activation of MAPK [187].

Moreover, in this model, vemurafenib did not alter the distribution or expansion of the adoptively transferred cells and also did not elevate the expressions of tumor antigens, which is indicating that the enhanced combined effect took place not because of a direct rise in the expressions of tumor antigens. Nonetheless, vemurafenib caused contradictory activation of MAPK that resulted in an elevated level of *in vivo* cytotoxic function and intratumoral cytokine release via adoptively transferred cells. In addition, the same mouse model was utilized to explore the role of alterations in chemokine signaling mediated via BRAF suppression in cancer cells and the combined activities with immune modifying antibody therapy [180]. It was also revealed that CD8^+^ T cells were partly needed for the antitumor function of BRAF inhibitors, which was found to be associated with the downregulation of tumor CCL2 generation after the oncogenic BRAF suppression. Combined therapy with agonistic anti-CD137 antibodies and BRAF inhibitors showed marked antitumor properties [180].

PI3K/AKT/mTOR signaling pathway is another important target for anticancer drug development because of its important contribution in the oncogenic signaling pathway in several histologies. Drug candidates that block this signaling at different stages are in clinical development and mammalian target of rapamycin (mTOR) inhibitors obtained approval for use in advanced RCC (aRCC) patients. Moreover, mTOR plays a crucial role in controlling the activities of immune cells, and inhibitors of mTOR are utilized for suppression of the immune system after organ transplantation. Nevertheless, suppression of mTOR signaling pathway has contradictory immune-stimulating activities, which can lead to the production of long-lived memory CD8^+^ T cells [[188], [189], [190]]. Extra useful activities of mTOR suppression were detected with hematopoietic stem cells and DCs, which is indicating that mTOR inhibitors might function via triggering promising immune cell alterations beyond their direct oncogene-targeting activities in cancer cells [191]. Additional use of targeted inhibitors of the PI3K/AKT/mTOR signaling is associated with the expressions of the immune suppressive membrane receptor PD-L1, which might have substantial uses for PD-1/PD-L1 blockade therapy. Phosphatase and tensin homolog (PTEN) loss can lead to PI3K pathway activation, which is a commonly seen event in glioblastoma multiforme (GBM). In addition, deficiency of PTEN within GBM cells was found to be linked with elevated PD-L1 expression and resulted in immune evasion, which might be reversed with inhibitors of PI3K [192]. PI3K inhibitors in combination with agonists of Toll-like receptor (TLR) were studied in a preclinical model, which showed better-combined activities against various murine tumors. Collectively, these beneficial properties were facilitated via the certain increase of polyfunctional T responses releasing IL-17 and IFN-γ [193].

Imatinib and various other c-kit and ABL inhibitors were found to successfully treat chronic myelogenous leukemia and gastrointestinal stromal tumors (GIST). Various studies have indicated that part of this success might be facilitated via triggering antitumor immune responses. Imatinib might also have off-target properties on immune effectors, regardless of its functions on tumor cells. Indeed, this activity was facilitated via the activation of promising cross-talk between NK cells and DCs [194,195]. The antitumor property of imatinib was found to be lost along with the depletion of CD8^+^ T cells and was increased via the blockade of CTLA-4 with monoclonal antibody therapy in a mouse model of spontaneous GIST development in transgenic mouse models carrying activating *KIT* mutation [196]. Moreover, in this model, imatinib decreased the expressions of the immune suppressive enzymes indoleamine 2,3-dioxygenase (IDO) via GIST tumor cells, which further mediated an increased level of the immune responses. In a different mouse model, the therapeutic function of dasatinib (a tyrosine kinase inhibitor) was significantly increased via immune induction with agonist anti-OX40 antibody therapy [197].

## 6 Challenges for combination approaches

Targeted therapies are highly effective in increasing responses with immunotherapy, however proper sequencing, dosage and timing of these agents will possibly be important for the success of combinatorial techniques. In addition, some targeted therapies containing immunostimulatory effects show immunosuppressive functions. For instance, even though inhibitors of HSP90 might elevate CTL-induced tumor cell lysis via increased antigen presentation on MHC class I molecules, they might also reduce some DCs and macrophage effects [198,199]. In a similar manner, temsirolimus (an mTOR inhibitor) might reduce the capacity of DCs to induce tumor-specific T cells [200], while HDAC inhibitors and bortezomib might hinder the activities of some NK cells [[201], [202], [203]]. Another important factor for combinatorial therapies is whether increased antitumor activities can be obtained without a corresponding rise in the level of severe toxicities. Inflammatory pathologies are major adverse events observed with some immunomodulatory agents including PD1 antibodies and ipilumimab, and combining therapies that influence different stages in immune responses may be expected to raise the chance of these side effects. Even though preclinical studies exploring these toxicities are very important, they might not completely anticipate the range of pathology that is seen in humans. For instance, inflammation of the pituitary gland is a common side effect of ipilimumab, however it was not predicted from experimental studies in mouse models [204]. Some preclinical studies also increased the chance that combination therapies including a 4-1BB agonist antibody and CTLA4 blockade may reduce instead of exacerbation of inflammatory toxicities [205].

## 7 Nursing and patient education on immunotherapy

Patients need continuous education regarding their clinical needs [206]. Therefore, individualized patient education needs to be provided by nurses including learning methods and consideration of barriers. Detection of irAEs by the patients and caregivers will permit the prompt and early intervention of irAEs, which is crucial for effective outcomes and continuance of therapy [207]. Overlooked symptoms linked with immunotherapy can result in devastating consequences for the health of patients, such as potentially life-threatening outcomes and permanent disability. Nurses need to be committed and competent to deliver useful educational opportunities to confirm quality and safe patient care [208]. Continuous research in the oncology field is leading to the development of new therapies. Thus, as new therapies come out, nurses are always at the front line of educating caregivers and patients while providing compassionate and holistic care [206]. Immunotherapy might or might not be the first line of treatment for patients. Therapies are customized by the types and stages of cancers. For patients receiving immunotherapies as their first, second or third line of therapy, it is important for caregivers and patients to appreciate and comprehend the differences between immunotherapy and cytotoxic chemotherapy. Immunotherapies target the stimulation of anticancer immune responses, while cytotoxic drugs interfere with DNA synthesis and cell division.

**Fig. 3:**
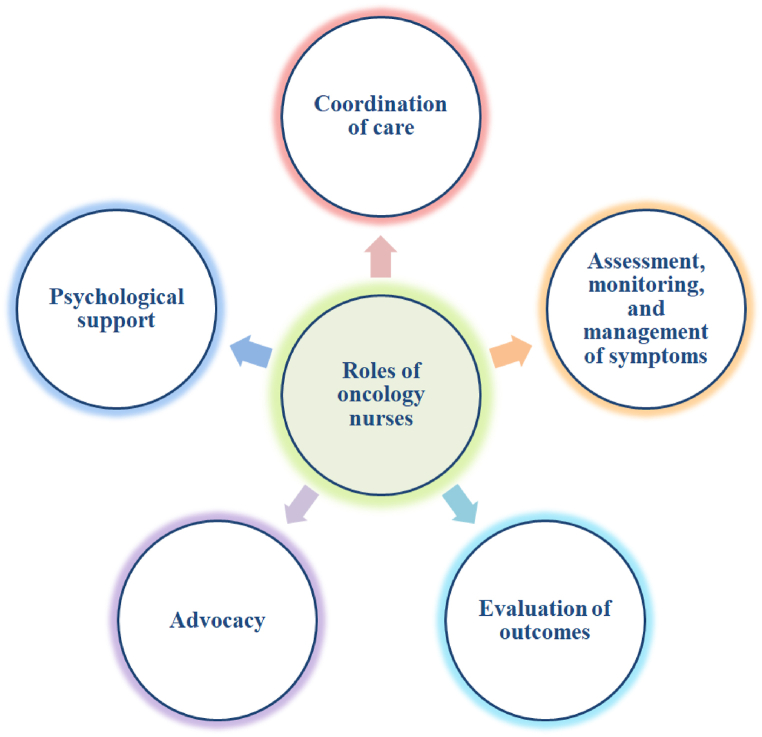
Roles of nurses in cancer treatment.

The effectiveness of cytotoxic chemotherapies relies on tumor shrinkage, while in individuals receiving immunotherapies, new lesions might develop or metastasis might grow prior to the reduction in total tumor burden owing to the infiltration of lymphocytes into tumors. When teaching patients on immunotherapy, an important teaching point is that immunotherapy-associated side effects take place due to dramatically different mechanisms as compared to cytotoxic chemotherapy-associated adverse events. Even though the presenting symptoms might be similar, knowledge regarding the mechanisms of action of CAR T-cell therapy, ICIs, and oncolytic viral therapies is important in comprehending the toxicity profiles and using different techniques to minimize or reverse their effect [209]. One analogy that can be utilized to elucidate the mechanisms of action of these drugs and difficult immunologic theories is to equate the immune system of patients to a car. For example, depressing the accelerator in a car (equivalent to activating T cells) is important for a car to move forward, which is comparable to an effective immune response against the tumor. Providing this analogy will help patients to better understand the mechanism of action of drugs and are frequently motivated by the idea that their own body is fighting against cancer [210]. Moreover, nurses have a significant contribution in understanding their therapeutic options, evaluating the knowledge of patients, and supporting while patients consider these treatment options (Fig. 3).

Creating strong relationships between caregivers and patients can encourage honest and open communication. Thus, it is crucial to emphasize that new symptoms always need to be reported to the practitioner. This education will be particularly useful for patients who might have doubts in reporting a change because of the fear that treatment might be discontinued. In addition, they need to know that continuation of a certain treatment with associated side effects might lead to more severe toxic effects and treatment might be on hold but not discontinued [211]. Patients ought to be educated that irAEs might take place at any time after the initiation of therapy or even after several months [211]. Verbal instruction is another important step in patient education [212]. However, verbal instruction should not be the only source of patient education and need to be associated with educational guides including written instructions. Furthermore, instructional guides must include and specify how and when to report any symptoms. Nurses also need to provide the patient with segmented and shorter education prior to moving on to another topic, if a patient struggles with any content [212]. Caregivers need to be involved in the educational process, since they can provide support to the patients through their duration of treatment and are reliable in mediating effective patient education. Moreover, this permits the patients to follow along with the verbal instructions. The teach-back method is a useful method to ensure whether the patients have successfully retained the information or not. This method also helps the nurses in assessing the level of patient knowledge regarding educational instructions via asking the patients to respond and confirm their understanding, using their own words [206].

For instance, nurses can ask the patients to restate the steps regarding what to do if diarrhea takes place. This method will ensure effective education and offer chances for clarification of misinterpretations. The teaching methods need to change along with the change in therapies. For instance, visual applications, web-based patient portals, and telemedicine give nurses remote access while increasing communication to further ameliorate the management of symptoms of patients via elevating the opportunity for educational experiences [213]. Telemedicine can enable patients living in rural areas far from healthcare centers to receive timely management [213]. Although various opportunities are growing, still nurses should cater to learner preferences, the age of the learner, and evaluate whether the methods are appropriate for the content or not. Nurses also should be attentive in understanding how patients are retrieving treatment information in a technologically advanced healthcare system. The internet has transformed how medical knowledge is exchanged between patients and nurses by permitting patients access to several forms of information [214]. Furthermore, patients use Internet resources as an approach to learn regarding their diseases and therapies [215]. Patients have access to numerous social media platforms, however the safest and best information comes from the healthcare team. Various other trustworthy sources for information come from drug manufacturers, peer-reviewed journals, and organization websites. It is important to advise patients against looking for clarification on treatment-associated side effects from social media platforms [206].

## 8 Nursing and patient education on targeted therapies

Cancer treatment was previously disease-focused and was dependent on a pathologic diagnosis. Nowadays, therapeutic choices are dependent on the findings of molecular testing and targeted therapies are focused on certain mutations along the signaling pathways. Therefore, nurses need to have knowledge regarding the genetic mutations and signaling mechanisms that regulate the growth of cancer cells to comprehend the processes of targeted therapies. Nurses can identify the structures of MoAbs, the type of targeted therapies, and the target of the agent in the names of these agents. This knowledge can be used in symptom management and patient education that is specific to targeted therapies. In addition to the new information emergence regarding signaling mechanisms and newly identified actionable targets, more therapeutic agents will be studied and permitted for clinical uses. The order of utilizing therapeutic agents as resistance develops must be clarified to patients. Patients need to know and adhere to the regimens for maximum efficacy, when combinations of agents are utilized. In order to ensure patient safety and education, oncology nurses ought to continue to learn about each development [17]. It is challenging to provide care to patients taking oral targeted therapies owing to the shifting paradigm of monitoring cancer patients from traditional office visits during intravenous treatment to more periodic observation with oral administration. Therefore, patients have a greater level of responsibility in self-management. However, systemic therapy can be distressful for numerous patients [216].

This stress can exert negative effects on the capacity of patients to remember and understand educational content and is linked with poor health outcomes. Oncology nurses have a significant role in educating patients on the integration and management of cancer therapy as well as how to live with cancer. Indeed, nurse-led patient education can decrease stress and ameliorate patients’ level of understanding and knowledge regarding treatment [217]. In addition, patients require detailed instructions on treatment adherence, optimal dosing, the incidence of follow-up assessments and their accountability in setting up the delivery of refills to mediate safety, usage of supportive care agents, reporting and monitoring side effects, potential food and drug interactions, administration and safe handling of medications. Each oral anticancer agent might have specific administration guidelines. Moreover, foods can have substantial effects on the tolerance and bioavailability of numerous targeted therapies for the treatment of cancer. For example, first-generation EGFR inhibitors need to be administered on an empty stomach (at least 1 h before eating or 2 h after eating) and patients ought to be warned to avoid Seville oranges and grapefruit juice since they can increase the bloodstream level of targeted therapies and result in more extended, significant side effects. On the other hand, some targeted therapies need to be administered with water and food to minimize esophagitis and ameliorate bioavailability. Nurses need to have a better understanding regarding the unique administration instructions of targeted therapies so that patients can be appropriately advised [218].

Nurses should adapt both spoken and written education to the patients' language and level of health literacy [219]. Furthermore, nurses ought to involve caregivers in teaching and utilizing interpretation services for patients for whom English is a second language. The teach-back method is also useful in the case of targeted therapies which inspires patients to enthusiastically participate in the education via asking questions that can help nurses to evaluate patient understanding and learning [219]. Moreover, nurses might help with setting expectations setting, such as normalizing dose reductions or dose interruptions to manage toxicities, the timing of repeat liquid or solid biopsies to evaluate for acquired resistance mutations during the time of progression, and the continuance of drug past progression if still obtaining clinical benefit. Interestingly, the Multinational Association of Supportive Care in Cancer (MASCC) developed an effective MASCC Oral Agent Teaching Tool (MOATT) with the input of a pharmacist, nurse coordinators, and nurse experts to improve health professional, caregiver, and patient education surrounding these possible life-extending but toxic therapies. MOATT identifies that concurrent illness, culture, and age, as well as social and family support might affect education needs. Indeed, patient education is an ongoing and dynamic process and might involve the usage of authorized tools and reminder cues, brochures, and asking to provide feedback in order to ameliorate application and information retention. MOATT is now available in 12 languages and endorses that education sessions should be structured to evaluate the patient's comprehension regarding the information presented via questioning, review drug-specific instructions such as potential interactions, side effects, schedule, and doses, offer general information on remembering, disposal, handling, and storage to administer oral cancer therapies and how to report side effects, and assess understanding of patients regarding their diseases, current medications, treatment plan, and capacity to obtain and administer an oral cancer therapy [218,220].

## 9 Future directions

In terms of educating adult cancer patients receiving immunotherapy treatments, it is very important for these patients to learn and apply the newly learned teachings to their care. Indeed, immunotherapy and targeted therapy are quite new to nurses, caregivers, and patients. Therefore, it is crucial for nurses to understand the treatment plans, associated risks and side effects to provide care to patients on immunotherapy as well as targeted therapy and how these therapies are different from other standards of care. Moreover, a continuous collaboration is required between the nurses and caregiver/patient [206].

## 10 Conclusion

Continuous research in the field of cancer treatment has greatly increased cancer and the human immune system. A range of clinically effective immunotherapies and targeted therapies are now available in cancer treatment. The availability of these therapies has significantly increased the options of cancer treatment, particularly for chemorefractory patients. Nurses associated with the care of cancer patients can obtain benefits from an improved understanding of targeted therapies and immunotherapies, their efficacy profile, their mechanisms of action, as well as education of cancer patients. Moreover, owing to the variations in the mechanisms of action of the main groups of cancer immunotherapy and targeted therapy, as well as the heterogeneity of AEs, it is very important that nurses are familiar with the different AEs so that they can properly assess and manage those AEs and ameliorate patient outcomes. This approach will also result in an improved understanding of why certain patients develop AEs and how they can be anticipated and alleviated in cancer patients.

## Funding

This research was funded by Deputyship for Research & Innovation, Ministry of Education in Saudi Arabia (ISP22-8).

## CRediT authorship contribution statement

**Hamad Ghaleb Dailah:** Supervision, Data curation, Conceptualization. **Abdullah Abdu Hommdi:** Formal analysis, Conceptualization. **Mahdi Dafer Koriri:** Data curation, Conceptualization. **Essa Mohammed Algathlan:** Writing – original draft, Data curation, Conceptualization. **Syam Mohan:** Writing – original draft, Supervision, Formal analysis, Conceptualization.

## Declaration of competing interest

The authors declare the following financial interests/personal relationships which may be considered as potential competing interests:The corresponding author of this manuscript is one of the associate editors If there are other authors, they declare that they have no known competing financial interests or personal relationships that could have appeared to influence the work reported in this paper.
